# Sleep Disorders: Is the Trigemino-Cardiac Reflex a Missing Link?

**DOI:** 10.3389/fneur.2017.00063

**Published:** 2017-02-27

**Authors:** Tumul Chowdhury, Barkha Bindu, Gyaninder Pal Singh, Bernhard Schaller

**Affiliations:** ^1^Department of Anesthesiology and Perioperative Medicine, University of Manitoba, Winnipeg, MB, Canada; ^2^Department of Neuro-anaesthesiology and Critical Care, All India Institute of Medical Sciences, New Delhi, India; ^3^Department of Research, University of Southampton, Southampton, UK

**Keywords:** trigemino-cardiac reflex, sleep apnea, bruxism, bradycardia, diving reflex

## Abstract

Trigeminal innervated areas in face, nasolacrimal, and nasal mucosa can produce a wide array of cardiorespiratory manifestations that include apnea, bradypnea, bradycardia, hypotension, and arrhythmias. This reflex is a well-known entity called “trigemino-cardiac reflex” (TCR). The role of TCR is investigated in various pathophysiological conditions especially in neurosurgical, but also skull base surgery procedures. Additionally, its significance in various sleep-related disorders has also been highlighted recently. Though, the role of diving reflex, a subtype of TCR, has been extensively investigated in sudden infant death syndrome. The data related to other sleep disorders including obstructive sleep apnea, bruxism is very limited and thus, this mini review aims to investigate the possible role and correlation of TCR in causing such sleep abnormalities.

## Introduction

Sleep disorders are a common increasing health problem in today’s industrialized world and can have a significant impact on quality of life and of working. They commonly manifest as excessive daytime sleepiness, difficulty initiating or maintaining sleep, or abnormal movements, behaviors, and sensations occurring during sleep. Sleep bruxism, thought to be a more intense form of rhythmic masticatory muscle activity (RMMA), has a prevalence of about 8% (1). Sleep apnea syndrome affects up to 3–5% of the adult human population. Unfortunately, the majority of sleep disorders remain undiagnosed to a large extent. Young et al. in 1997 reported that 80–90% of adults with clinically significant sleep-disordered breathing remain undiagnosed (2).

In this regard, the role of the trigemino-cardiac reflex (TCR) is never extensively explored. The TCR is one of the most powerful autonomic reflexes of the body that helps reduce heart rate under challenging situations by acting as oxygen-conserving reflex (3–5). The trigeminal nerve can be stimulated anywhere along its course and causes sympathetic withdrawal and parasympathetic over activity through the vagus nerve resulting in bradycardia or even asystole, apnea, bradypnea, and hypotension. Various manifestations of the TCR include the naso-cardiac reflex, peripheral TCR, the diving reflex (DR), and the central TCR (6–10). Interestingly, DR, a subtype of TCR, has been hypothesized to have a role in sudden infant death syndrome (SIDS) (11) and the TCR is also linked to sleep disorders like sleep-related bruxism (SB) (12). It is reported that sudden microarousals (MA) occurring in the brain due to airway obstruction during sleep cause tachycardia, which stimulates RMMA and teeth grinding that activate the TCR resulting in bradycardia. The physiological basis and importance of conditions like sleep bruxism and obstructive sleep apnea (OSA) are still not completely understood. This is a narrative mini review and aims to provide facts and hypotheses that the TCR plays a central role in various sleep disorders.

## Normal Sleep

About one-third of our lives are spent sleeping. Two types of sleep have been described: non-rapid eye movement (NREM) and rapid eye movement (REM). NREM further has four stages, 1, 2, 3 and 4, representing a continuum of relative depth of sleep. NREM and REM cycle throughout the night. Normal individuals first enter sleep in NREM, which progresses through stages 1, 2, 3 and 4, and then enter REM sleep. NREM sleep occupies 75–80% of sleep and REM sleep accounts for 20–25%. The average length of NREM–REM cycles is 70–100 min initially and later increases to 90–120 min as sleep progresses (13). The duration of REM sleep in each cycle increases as the night progresses.

The four stages of NREM sleep have characteristic brain physiology. Stage 1 accounts for 2–5% of total sleep and gets easily disrupted by loud noise. EEG waves in this stage show transition from alpha waves to low voltage, mixed frequency waves. Stage 2 accounts for 45–55% of total sleep and is characterized by low voltage, mixed frequency waves with sleep spindles and K-complexes. Stages 3 and 4, together called slow-wave sleep, are characterized by high voltage, slow wave activity. Stage 3 accounts for 3–8% and stage 4 for 10–15% of total sleep. Among all stages of NREM sleep, arousal threshold is highest for stage 4 (13). REM sleep is characterized by theta waves and slow alpha waves, muscle atonia, and bursts of REMs (13). Most of dreaming and memory consolidation occur during REM sleep (14).

Non-rapid eye movement and REM sleep vary considerably concerning physiological changes (15, 16). Broadly, brain activity, heart rate, blood pressure, cerebral blood flow, and respiration decrease during NREM and increase in REM sleep. Muscle tone is absent, and body temperature regulation is disturbed during REM sleep and sexual arousals occur more frequently in REM sleep. Airway resistance increases during both NREM and REM sleep, compared to wakefulness (17).

## Sleep Disorders

Around 90 different sleep disorders have been identified so far. The third edition of International Classification of Sleep Disorders (ICSD-3) classifies sleep disorders into seven major diagnostic sections—insomnia, sleep-related breathing disorders, central disorders of hypersomnolence, circadian rhythm sleep–wake disorders, parasomnias, sleep-related movement disorders, and other sleep disorders (18). The ICSD-3 classifies OSA as a sleep-related breathing disorder while SB is classified as a sleep-related movement disorder. OSA, usually occurs due to mild to severe collapse of the airway (mainly obstruction by soft tissues) in up to 9% of women and 24% of men (19, 20); while the RMMA is much more widespread and occurs in up to 60% of normal population, 80% of these occurring in NREM sleep (21).

While insomnia is defined as sleep initiation or maintenance problem despite adequate circumstances to sleep and having daytime consequences, sleep-related breathing disorders include OSA, central sleep apnea syndromes, sleep-related hypoventilation disorders, and sleep-related hypoxemia disorder. The diagnosis of OSA in adults requires either presence of signs/symptoms or associated medical/psychiatric history coupled with five or more obstructive respiratory events per hour of sleep. Alternatively, OSA is also diagnosed based on ≥15 obstructive respiratory events per hour, even in the absence of associated symptoms or disorders (18). Central disorders of hypersomnolence are characterized by excessive daytime sleepiness that cannot be attributed to another sleep disorder or abnormalities of circadian rhythm and is often caused by intrinsic CNS abnormalities that control the sleep–wake cycle. Circadian rhythm sleep–wake disorders are defined as a chronic or recurrent pattern of sleep–wake rhythm disruption lasting for at least 3 months. Parasomnias can be either NREM related or REM related and include conditions such as sleep walking, nightmare disorder, sleep enuresis, sleep-related hallucinations, etc. Sleep-related movement disorders are characterized by simple, often stereotyped movements during sleep and include restless legs syndrome, periodic limb movement disorder, SB, benign sleep myoclonus of infancy, etc. SB refers to RMMA characterized by tooth grinding or clenching in sleep that lacks a definitive physiological purpose and is associated with intense sleep arousal activity (22). It is polysomnographically characterized by forceful, short (approximately 250 ms) rhythmic, or prolonged contractions of masticatory muscles (23).

The etiology of sleep disorders can be related to social, psychological, and anatomical factors. Insomnia occurs because of a combination of biological, mental, and social factors, but, stress, old age, and female gender play a major role. OSA occurs due to frequent periods of collapse of the pharyngeal airway. This causes a reduction in oxygen saturation of blood leading to cortical and brainstem arousals. Risk factors for OSA include obesity, male sex, alcoholism, increasing age, etc., and it has been found to be associated with higher incidence of hypertension, myocardial infarction, congestive heart failure, and diabetes (24–27). Narcolepsy and cataplexy have been found to be involved in the presence of HLA-DQB1*0602 haplotype and loss of hypocretin (orexin) producing neurons in the brain (28). The SIDS, a sudden death of infants less than a year old during sleep, is currently the third leading cause of death in infants in the United States (29). The exact cause is still not known but developmental abnormalities of the cardiorespiratory system are one of the proposed etiologies (30). SB can occur due to both central (involving brain neurotransmitters, basal ganglia, limbic system) (31) and peripheral (dental occlusion or other morphological features of jaw system) factors, with central factors being more important (32). Patients of sleep bruxism, a more intense form of RMMA, experience higher episodes of RMMA per hour than patients without bruxism (13). Three types of bruxism have been described: tooth grinding with friction sounds, tooth clenching, and tapping or jaw bracing (33).

## Linkage of TCR to Various Sleep Disorders

**Figure 1:**
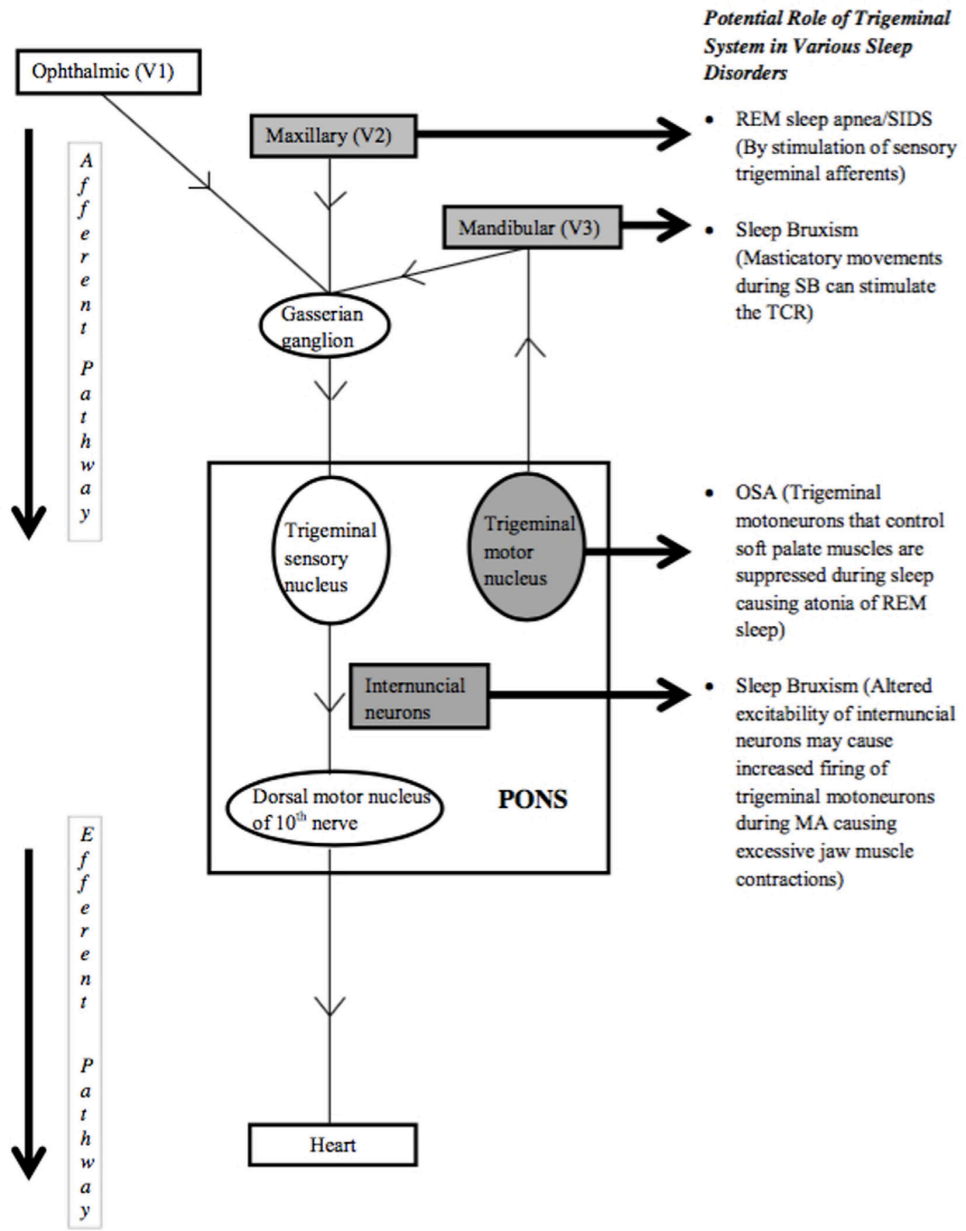
**Trigemino-cardiac reflex pathway and sleep disorders**.

The TCR, as the most powerful autonomic reflex, is known to cause bradycardia and apnea. The resulting decrease in heart rate and apnea are the mechanisms through which the TCR can be implicated in causing various sleep disorders (Figure 1). In this regard, the role of peripheral TCR (DR) in causing SIDS has been investigated (6, 11). The rostral trigeminal sensory nuclear complex neurons convey information from orofacial regions to the thalamus. Cairns et al. have reported suppression of these neurons during active sleep, the exact cause of which is not known, but, is speculated to contribute to maintaining the integrity of active sleep (34). Classical cardiorespiratory changes (bradycardia, apnea, and hypertension) associated with OSA are multifactorial; however, the role of peripheral TCR (DR) in causing such changes cannot be underestimated (35). Interestingly, the TCR can also be linked to both the causation as well as systemic manifestations of OSA. One of the key components of OSA is hypoxemia that itself acts as a potential risk factor for inciting the TCR. Also, hypoxemia is a known cause of sudden death in such patients; therefore may suggest the role of the TCR in victims of sudden death as well (35). Recently, the role of TCR is postulated for the phenomenon of sleep bruxism and thus, the TCR seems to cause a broad range of sleep disorders that are elaborated below in detail.

## Sleep Bruxism

Heart rate remains stable during normal sleep when breathing is normal. However, when breathing becomes labored due to airway obstruction, the fall in oxygen content of blood causes the body to put extra effort to obtain oxygen, leading to MA of the brain. MA episodes are characterized by tachycardia, increased muscle tone, and increased brain activity, while the person remains asleep (36). Sleeping in the supine position also seems to affect the frequency of SB, probably because this position is associated with airway obstruction (37). Hypotheses postulated for RMMA–SB episodes include a need to increase salivary flow for lubrication during sleep, need to reduce heart rate during MA of the brain, and need to open the airway during episodes of airway collapse (38, 39). Schames et al. in 2012 discussed the physiology of SB and the TCR as a probable cause of SB. The authors have discussed how SB occurs as a result of tachycardia during MA and then stimulates a vagal response (12). SB has been reported to be secondary to MA of the brain earlier by Kato et al. in 2001 (40). A sequence of physiological changes starting with increased respiratory rate, followed by increased EEG activity and an increase in heart rate has been described to occur just before an RMMA episode (41). Schames et al. proposed that tachycardia occurs due to brain MA and probably causes an RMMA–SB episode. Whereas, masticatory movements stimulate the TCR and result in bradycardia, teeth contact occurring during SB serves as an even stronger stimulus for the TCR resulting in more profound bradycardia than RMMA alone (12). Thus, RMMA–SB episodes have been proposed to be an auto-regulatory process occurring during sleep with TCR playing a central role in SB. The fact that partial masticatory movements, as in the submaximal opening of mouth by a spring device, causes prolonged reduction of blood pressure and heart rate has been substantiated by Brunelli et al. (42).

Chase et al. identified neurons in the medullary reticular formation to be responsible for the postsynaptic inhibition of trigeminal motor neurons during active sleep, causing atonia of masseter muscles (43). Another report by Gastaldo et al. suggests the presence of a group of interneurons that modulate the trigeminal motor system. Alteration in the excitability of this group of interneurons could increase the firing probability in trigeminal motor neurons during sleep arousals leading to excessive jaw muscle contractions, as seen in SB (44).

Though the physiology of SB is not exactly known, this above-mentioned available knowledge does point toward the TCR playing an important role in its pathogenesis, but, will need further confirmatory evidence in implicating TCR definitively.

## OSA, Central Sleep Apnea, Sudden Death, and SIDS

Noradrenergic cells in the brainstem are known to project to trigeminal motoneurons which control soft palate muscles, and their discharge activity has been positively correlated with sleep state-dependent changes in muscle tone (45). Schwarz et al. in 2008 demonstrated that noradrenaline plays a modulatory role in potentiating glutamate-dependent synaptic transmission (46). The same authors in 2010 reported that noradrenaline could not trigger motoneuron excitability on its own; instead, it acts to facilitate glutamatergic motor excitation. The glutamatergic drive is reported to be minimal during REM sleep causing the atonia of REM sleep (47), the reason why drugs that increase noradrenergic neurotransmission have had limited success in increasing muscle tone during REM sleep (48). Schwarz and Peever propose that drugs that boost glutamate receptor function in conjunction with noradrenergic agents could be successful in counteracting sleep-related motor suppression, such as that underlying OSA (49). So, the trigeminal system seems to have a role in OSA as well, but whether the TCR is involved or not, needs to be explored.

The naso-trigeminal reflex, a form of peripheral TCR, is known to be a protective response for the upper airways from noxious substances. Dutschmann and Herbert in 1999 tested the hypothesis that stimulation of sensory trigeminal afferents might contribute to REM sleep apnea. They reported that injection of carbachol (mixed agonist for nicotinic and muscarinic acetylcholine receptors) into pontine reticular nuclei of anesthetized rats causes marked potentiation of ethmoidal nerve induced respiratory depression and induces REM sleep like respiratory suppression, even apnea in some cases. The authors speculated that activation of sensory trigeminal afferents during REM sleep could easily trigger centrally mediated apneas and cause pathological conditions like REM sleep apnea or SIDS (50). An increase in upper airway resistance and increased nasal discharge, as seen in allergic rhinitis and rhino sinusitis, have been found responsible for disordered breathing in sleep and MA (51). Tobacco smoke causes congestion and increased nasal airflow resistance. Trigeminal neurons can be activated by mast cell mediators and may contribute to sneezing and itching (52). Trigeminal fibers to the central nervous system convey the sensation of nasal pruritus. The stimulation of nasal trigeminal receptors by factors such as nasal congestion, nasal discharge, or smoke might activate the TCR and may cause sleep disorders. Allergic rhinitis is known to cause neuronal hyper-responsiveness of upper airways to stimuli that activate nasal afferents (53). Nasal inhalation of particulate material or rubbing of inferior turbinate has been shown to cause bronchoconstriction and cardio-depression, through stimulation of trigeminal afferents and activation of TCR (54). A similar response to nasal congestion or nasal discharge by activation of TCR or DR may be caused in allergic rhinitis. Lavie et al. have suggested that increased upper airway resistance and nasal discharge seen in allergic rhinitis cause disordered breathing in sleep and MA (up to 10 times more than in normal controls) (51). Whether these MA episodes are associated with higher incidence of SB in patients of allergic rhinitis needs to be established. Cook et al. observed an exaggerated response to cold stimulus applied on face (simulating DR) in people with non-eosinophilic non-allergic rhinitis (NENAR) as compared to normal individuals (55). There was a significant increase in airway resistance in patients of NENAR due to increase in parasympathetic tone [autonomic control of nasal vasculature (56)] but not in normal individuals. Here, the afferent is mediated by the trigeminal nerve while the efferent limb is parasympathetic. This study observed an exaggerated DR or TCR in individuals with NENAR and thus there may be a possible association of nasal discharge or congestion and sleep disorders linked through TCR in such individuals. This needs to be explored further. It is well known that OSA may occur in patients with rhinitis and therefore, sleep disorders like OSA or SB might be linked *via* activation of TCR by nasal congestion/discharge or inflammatory triggers. Further research in this direction is warranted.

Heiser et al. have demonstrated that trigeminal stimulation during sleep leads to arousals in a dose- and time-dependent manner (57, 58). Several authors have shown earlier that failure to arouse from sleep could be the causative factor for SIDS. Decreased spontaneous arousals during sleep in SIDS victims compared with control infants has been described (59, 60), and has been attributed to the possible immaturity of the autonomic nervous system as shown by Tuladhar et al. in their study, where they examined heart rate responses to arousing and non-arousing trigeminal stimuli (61). Tuladhar et al. in 2005 also reported that the bradycardia occurring in response to non-arousing stimulation of the trigeminal nerve is present in infants up to 6 months of age and is stronger when sleeping in the supine position and the NREM (quiet sleep) sleep stage (62).

It is a well-established fact that the autonomic nervous system plays a critical role in the pathogenesis of various cardiac arrhythmias (63, 64). For example, atrial fibrillation reportedly has an association with an imbalance between the sympathetic and parasympathetic supply of the heart (65). Similarly, ventricular fibrillation has been shown to be initiated by sympathetic stimulation, especially in an ischemic heart (66). Though sinus arrhythmia is considered physiological during sleep and bradyarrhythmias also can occur due to increased vagal activity (67), especially during NREM sleep, the increased sympathetic drive at the end of sleep can cause adverse events during awakening from sleep (68). Sudden cardiac death occurring due to ventricular arrhythmias, especially ventricular fibrillation, carries a mortality rate of up to 250,000–450,000 per year in the United States (69). OSA-associated hypoxemia results in bradycardia and increased peripheral sympathetic activity resulting in vasoconstriction (70), the same response that occurs during DR. A direct relationship between the severity of OSA and the risk of sudden cardiac death at night has been proposed, probably due to greater number of nocturnal ischemic events in these patients (71). In a recent study on more than 10,000 sleep study of patients, 78% were found to have sleep apnea and during the follow-up of 15 years, they found that 142 (2%) had sudden cardiac arrest, either fatal or resuscitated (72). Though there is no direct linking evidence between the TCR and sudden cardiac death, the amount of influence that the autonomic system exerts on the heart, manifesting either as arrhythmias or as OSA-induced bradycardia and hypertension, does suggest the possibility of the TCR playing a role in sudden death as well.

Based on these reports, the TCR does seem to have a role in various sleep disorders, either due to altered noradrenergic or glutamatergic control, or in the form of naso-trigeminal reflex, or as a result of the immaturity of the autonomic nervous system. It underlines again that the TCR is one of the most important phenomenologies in (clinical) neuroscience.

## Limitation

This review is not a systematic review. It is more hypothetical in nature and is aimed to postulate the role of TCR in various sleep disorders so that future research could be directed on this important topic.

## Conclusion

The pathophysiology of sleep disorders like OSA, SB, SD, and SIDS is not entirely understood at this time. Various hypotheses have been proposed for each of these conditions. The TCR might be playing a protective role in the case of sleep bruxism, while an exaggerated form of this reflex could be responsible for SD and SIDS. Based on available literature and exemplary cases, the TCR can be thought of as also playing an important role in various sleep disorders, though further evidence is warranted before it can be definitively implicated.

## Author Contributions

TC has made substantial contributions to conception and design, and/or acquisition of data, and/or analysis and interpretation of data, and helped in writing the manuscript. BB has participated in data acquisition and interpretation of data and writing the article. GS has participated in drafting and writing the article. BS has participated in developing the concept and writing. All the authors have given final approval for submission of this version.

## Conflict of Interest Statement

The authors declare that the research was conducted in the absence of any commercial or financial relationships that could be construed as a potential conflict of interest.

## References

[1] Reding GR Rubright WC Zimmerman SO Incidence of bruxism. J Dent Res (1966) 45:1198–204. 10.1177/00220345660450042701 5224088

[2] Young T Evans L Finn L Palta M. Estimation of the clinically diagnosed proportion of sleep apnea syndrome in middle-aged men and women. Sleep (1997) 20:705–6. 9406321 10.1093/sleep/20.9.705

[3] Sandu N Spiriev T Lemaitre F Filis A Schaller B Trigemino-Cardiac Reflex Examination Group (TCREG). New molecular knowledge towards the trigemino-cardiac reflex as a cerebral oxygen-conserving reflex. ScientificWorldJournal (2010) 10:811–7. 10.1100/tsw.2010.71 20454763 PMC5763844

[4] Schaller B Cornelius JF Sandu N Ottaviani G Perez-Pinzon MA. Oxygen-conserving reflexes of the brain: the current molecular knowledge. J Cell Mol Med (2009) 13:644–7. 10.1111/j.1582-4934.2009.00659.x 19438971 PMC3822871

[5] Sandu N Cornelius J Filis A Nöthen C Rasper J Kulinsky VI Cerebral hemodynamic changes during the trigeminocardiac reflex: description of a new animal model protocol. ScientificWorldJournal (2010) 10:1416–23. 10.1100/tsw.2010.136 20661534 PMC5763697

[6] Lemaitre F Chowdhury T Schaller B. The trigeminocardiac reflex – a comparison with the diving reflex in humans. Arch Med Sci (2015) 11:419–26. 10.5114/aoms.2015.50974 25995761 PMC4424259

[7] Meuwly C Golanov E Chowdhury T Erne P Schaller B. Trigeminal cardiac reflex: new thinking model about the definition based on a literature review. Medicine (Baltimore) (2015) 94:e484. 10.1097/MD.0000000000000484 25654391 PMC4602726

[8] Sandu N Sadr-Eshkevari P Schaller BJ Trigemino-Cardiac Reflex Examination Group (TCREG). Usefulness of case reports to improve medical knowledge regarding trigemino-cardiac reflex in skull base surgery. J Med Case Rep (2011) 5:149. 10.1186/1752-1947-5-149 21496216 PMC3089793

[9] Nöthen C Sandu N Prabhakar H Filis A Arasho BD Buchfelder M Trigemino-cardiac reflex and antecedent transient ischemic attacks. Expert Rev Cardiovasc Ther (2010) 8:509–12. 10.1586/erc.10.19 20397825

[10] Schaller B. Trigeminocardiac reflex. A clinical phenomenon or a new physiological entity? J Neurol (2004) 251:658–65. 10.1007/s00415-004-0458-4 15311339

[11] Singh GP Chowdhury T Bindu B Schaller B Sudden infant death syndrome – role of trigeminocardiac reflex: a review. Front Neurol (2016) 7:221 10.3389/fneur.2016.00221 27994573 PMC5136573

[12] Schames SE Schames J Schames M Chagall-Gungur SS. Sleep bruxism, an autonomic self-regulating response by triggering the trigeminal cardiac reflex. J Calif Dent Assoc (2012) 40:670–1, 674–6. 22953526

[13] Carskadon MA Dement WC Normal human sleep: an overview. 5th ed In: Kryger MH Roth T Dement WC, editors. Principles and Practice of Sleep Medicine. St. Louis: Elsevier Saunders (2005). p. 16–26.

[14] Smith C Lapp L. Increases in number of REMs and REM density in humans following an intensive learning period. Sleep (1991) 14:325–30. 1947596 10.1093/sleep/14.4.325

[15] Madsen PL Schmidt JF Wildschiødtz G Friberg L Holm S Vorstrup S Cerebral O_2_ metabolism and cerebral blood flow in humans during deep and rapid-eye movement sleep. J Appl Physiol (1991) 70:2597–601. 1885454 10.1152/jappl.1991.70.6.2597

[16] Somers VK Dyken ME Mark AL Abboud FM. Sympathetic-nerve activity during sleep in normal subjects. N Engl J Med (1993) 328:303–7. 10.1056/NEJM199302043280502 8419815

[17] Colten HR Altevogt BM, editors. Sleep physiology. Sleep Disorders and Sleep Deprivation: An Unmet Public Health Problem. Washington: National Academies Press (2006). p. 33–54. 20669438

[18] Sateia MJ. International classification of sleep disorders-third edition: highlights and modifications. Chest (2014) 146:1387–94. 10.1378/chest.14-0970 25367475

[19] Bixler EO Vgontzas AN Ten Have T Tyson K Kales A. Effects of age on sleep apnea in men: I. Prevalence and severity. Am J Respir Crit Care Med (1998) 157:144–8. 10.1164/ajrccm.157.1.9706079 9445292

[20] Bixler EO Vgontzas AN Lin HM Ten Have T Rein J Vela-Bueno A Prevalence of sleep-disordered breathing in women: effects of gender. Am J Respir Crit Care Med (2001) 163:608–13. 10.1164/ajrccm.163.3.9911064 11254512

[21] Lavigne GJ Rompre PH Poirier G Huard H Kato T Montplaisir JY. Rhythmic masticatory muscle activity during sleep in humans. J Dent Res (2001) 80:443–8. 10.1177/00220345010800020801 11332529

[22] Kato T Montplaisir JY Guitard F Sessle BJ Lund JP Lavigne GJ. Evidence that experimentally induced sleep bruxism is a consequence of transient arousal. J Dent Res (2003) 82:284–8. 10.1177/154405910308200408 12651932

[23] Lavigne GJ Rompré PH Montplaisir JY. Sleep bruxism: validity of clinical research diagnostic criteria in a controlled polysomnographic study. J Dent Res (1996) 75:546–52. 10.1177/00220345960750010601 8655758

[24] Young T Javaheri S Systemic and pulmonary hypertension in obstructive sleep apnea. 4th ed In: Kryger MH Roth T Dement WC, editors. Principles and Practice of Sleep Medicine. Philadelphia: Elsevier Saunders (2005). p. 1192–202.

[25] Marin JM Carrizo SJ Vicente E Agusti AG. Long-term cardiovascular outcomes in men with obstructive sleep apnoea-hypopnoea with or without treatment with continuous positive airway pressure: an observational study. Lancet (2005) 365:1046–53. 10.1016/S0140-6736(05)71141-7 15781100

[26] Javaheri S Parker TJ Liming JD Corbett WS Nishiyama H Wexler L Sleep apnea in 81 ambulatory male patients with stable heart failure. Types and their prevalences, consequences, and presentations. Circulation (1998) 97:2154–9. 10.1161/01.CIR.97.21.2154 9626176

[27] Punjabi NM Shahar E Redline S Gottlieb DJ Givelber R Resnick HE Sleep-disordered breathing, glucose intolerance, and insulin resistance: the Sleep Heart Health Study. Am J Epidemiol (2004) 160:521–30. 10.1093/aje/kwh261 15353412

[28] Peyron C Faraco J Rogers W Ripley B Overeem S Charnay Y A mutation in a case of early onset narcolepsy and a generalized absence of hypocretin peptides in human narcoleptic brains. Nat Med (2000) 6:991–7. 10.1038/79690 10973318

[29] CDC. Sudden Infant Death Syndrome (SIDS) [Online] (2006). Available from: http://www.cdc.gov/sids/data.htm

[30] Verrier RL Josephson ME Cardiac arrhythmogenesis during sleep: mechanisms, diagnosis, and therapy. 4th ed In: Kryger MH Roth T Dement WC, editors. Principles and Practice of Sleep Medicine. Philadelphia: Elsevier Saunders (2005). p. 1171–91.

[31] Lavigne GJ Kato T Kolta A Sessle BJ. Neurobiological mechanisms involved in sleep bruxism. Crit Rev Oral Biol Med (2003) 14:30–46. 10.1177/154411130301400104 12764018

[32] Lobbezoo F Naeije M. Bruxism is mainly regulated centrally, not peripherally. J Oral Rehabil (2001) 28:1085–91. 10.1046/j.1365-2842.2001.00839.x 11874505

[33] Vetrugno R Provini F Plazzi G Lombardi C Liguori R Lugaresi E Familial nocturnal facio-mandibular myoclonus mimicking sleep bruxism. Neurology (2002) 58:644–7. 10.1212/WNL.58.4.644 11865148

[34] Cairns BE Fragoso MC Soja PJ. Activity of rostral trigeminal sensory neurons in the cat during wakefulness and sleep. J Neurophysiol (1995) 73:2486–98. 7666154 10.1152/jn.1995.73.6.2486

[35] Ludka O Konecny T Somers V Sleep apnea, cardiac arrhythmias, and sudden death. Tex Heart Inst J (2011) 38:340–3. 21841855 PMC3147220

[36] Huynh N Kato T Rompré PH Okura K Saber M Lanfranchi PA Sleep bruxism is associated to micro-arousals and an increase in cardiac sympathetic activity. J Sleep Res (2006) 15:339–46. 10.1111/j.1365-2869.2006.00536.x 16911037

[37] Miyawaki S Lavigne GJ Pierre M Guitard F Montplaisir JY Kato T. Association between sleep bruxism, swallowing-related laryngeal movement, and sleep positions. Sleep (2003) 26:461–5. 12841373

[38] Thie NM Kato T Bader G Montplaisir JY Lavigne GJ. The significance of saliva during sleep and the relevance of oromotor movements. Sleep Med Rev (2002) 6:213–27. 10.1053/smrv.2001.0183 12531122

[39] Landry M Rompre PH Manzini C Guitard F de Grandmont P Lavigne GJ. Reduction of sleep bruxism using a mandibular advancement device: an experimental controlled study. Int J Prosthodont (2006) 19:549–56. 17165292

[40] Kato T Rompre PH Montplaisir JY Sessle BJ Lavigne GJ. Sleep bruxism: an oromotor activity secondary to micro-arousal. J Dent Res (2001) 80:1940–4. 10.1177/00220345010800101501 11706956

[41] Lavigne GJ Huynh N Kato T Okura K Adachi K Yao D Genesis of sleep bruxism: motor and autonomic-cardiac interactions. Arch Oral Biol (2007) 52:381–4. 10.1016/j.archoralbio.2006.11.017 17313939

[42] Brunelli M Coppi E Tonlorenzi D Del Seppia C Lapi D Colantuoni A Prolonged hypotensive and bradycardic effects of passive mandibular extension: evidence in normal volunteers. Arch Ital Biol (2012) 150:231–7. 10.4449/aib.v150i4.1420 23479456

[43] Chase MH Enomoto S Hiraba K Katoh M Nakamura Y Sahara Y Role of medullary reticular neurons in the inhibition of trigeminal motoneurons during active sleep. Exp Neurol (1984) 84:364–73. 10.1016/0014-4886(84)90233-4 6714349

[44] Gastaldo E Quatrale R Graziani A Eleopra R Tugnoli V Tola MR The excitability of the trigeminal motor system in sleep bruxism: a transcranial magnetic stimulation and brainstem reflex study. J Orofac Pain (2006) 20:145–55. 16708832

[45] Chan E Steenland HW Liu H Horner RL. Endogenous excitatory drive modulating respiratory muscle activity across sleep-wake states. Am J Respir Crit Care Med (2006) 174:1264–73. 10.1164/rccm.200605-597OC 16931636

[46] Schwarz PB Yee N Mir S Peever JH. Noradrenaline triggers muscle tone by amplifying glutamate-driven excitation of somatic motoneurones in anaesthetized rats. J Physiol (2008) 586:5787–802. 10.1113/jphysiol.2008.159392 18845613 PMC2655409

[47] Burgess CR Lai D Siegel J Peever J. An endogenous glutamatergic drive onto somatic motoneurons contributes to the stereotypical pattern of muscle tone across the sleep-wake cycle. J Neurosci (2008) 28:4649–60. 10.1523/JNEUROSCI.0334-08.2008 18448642 PMC6670438

[48] Horner RL. Respiratory motor activity: influence of neuromodulators and implications for sleep disordered breathing. Can J Physiol Pharmacol (2007) 85:155–65. 10.1139/y06-089 17487255

[49] Schwarz PB Peever JH. Noradrenergic control of trigeminal motoneurons in sleep: relevance to sleep apnea. Adv Exp Med Biol (2010) 669:281–4. 10.1007/978-1-4419-5692-7_57 20217366

[50] Dutschmann M Herbert H. Pontine cholinergic mechanisms enhance trigeminally evoked respiratory suppression in the anesthetized rat. J Appl Physiol (1999) 87:1059–65. 10484577 10.1152/jappl.1999.87.3.1059

[51] Lavie P Gertner R Zomer J Podoshin L Breathing disorders in sleep associated with “microarousals” in patients with allergic rhinitis. Acta Otolaryngol (1981) 92:529–33. 10.3109/00016488109133292 7315270

[52] Naclerio RM Bachert C Baraniuk JN. Pathophysiology of nasal congestion. Int J Gen Med (2010) 3:47–57. 10.2147/IJGM.S8088 20463823 PMC2866558

[53] Canning BJ. Neurology of allergic inflammation and rhinitis. Curr Allergy Asthma Rep (2002) 2:210–5. 10.1007/s11882-002-0021-2 11918862

[54] Baraniuk JN Merck SJ. Nasal reflexes: implications for exercise, breathing and sex. Curr Allergy Asthma Rep (2008) 8:147–53. 10.1007/s11882-008-0025-7 18417057 PMC4209300

[55] Cook JA Hamilton JW Jones AS The diving reflex in non-eosinophinic non-allergic rhinitis. Clin Otolaryngol Allied Sci (1996) 21:226–7. 10.1111/j.1365-2273.1996.tb01730.x 8818492

[56] Anggård A. Parasympathetic influence on the nasal mucosa. Acta Otolaryngol (1977) 83:22–4. 10.3109/00016487709128806 842322

[57] Heiser C Baja J Lenz F Sommer JU Hörmann K Herr RM Trigeminal induced arousals during human sleep. Sleep Breath (2015) 19:553–60. 10.1007/s11325-014-1046-1 25115885

[58] Stuck BA Stieber K Frey S Freiburg C Hörmann K Maurer JT Arousal responses to olfactory or trigeminal stimulation during sleep. Sleep (2007) 30:506–10. 10.1093/sleep/30.4.506 17520795

[59] Kahn A Groswasser J Rebuffat E Sottiaux M Blum D Foerster M Sleep and cardiorespiratory characteristics of infant victims of sudden infant death: a prospective case-control study. Sleep (1992) 15:287–92. 1519001 10.1093/sleep/15.4.287

[60] Schechtman VL Harper RM Wilson AJ Southall DP. Sleep state organization in normal infants and victims of the sudden infant death syndrome. Pediatrics (1992) 89:865–70. 1579396

[61] Tuladhar R Harding R Michael Adamson T Horne RS. Comparison of postnatal development of heart rate responses to trigeminal stimulation in sleeping preterm and term infants. J Sleep Res (2005) 14:29–36. 10.1111/j.1365-2869.2004.00434.x 15743331

[62] Tuladhar R Harding R Adamson TM Horne RS. Heart rate responses to non-arousing trigeminal stimulation in infants: effects of sleep position, sleep state and postnatal age. Early Hum Dev (2005) 81:673–81. 10.1016/j.earlhumdev.2005.04.002 16039075

[63] Harris AS Estandia A Tillotson RF Ventricular ectopic rhythms and ventricular fibrillation following cardiac sympathectomy and coronary occlusion. Am J Physiol (1951) 165:505–12. 14846969 10.1152/ajplegacy.1951.165.3.505

[64] Shen MJ Zipes DP. Role of the autonomic nervous system in modulating cardiac arrhythmias. Circ Res (2014) 114:1004–21. 10.1161/CIRCRESAHA.113.302549 24625726

[65] Bettoni M Zimmermann M. Autonomic tone variations before the onset of paroxysmal atrial fibrillation. Circulation (2002) 105:2753–9. 10.1161/01.CIR.0000018443.44005.D8 12057990

[66] Opthof T Misier AR Coronel R Vermeulen JT Verberne HJ Frank RG Dispersion of refractoriness in canine ventricular myocardium. Effects of sympathetic stimulation. Circ Res (1991) 68:1204–15. 10.1161/01.RES.68.5.1204 2018987

[67] Verrier RL Josephson ME Impact of sleep on arrhythmogenesis. Circ Arrhythm Electrophysiol (2009) 2:450–9. 10.1161/CIRCEP.109.867028 19808502 PMC2744995

[68] Muller JE Ludmer PL Willich SN Tofler GH Aylmer G Klangos I Circadian variation in the frequency of sudden cardiac death. Circulation (1987) 75:131–8. 10.1161/01.CIR.75.1.131 3791599

[69] Lloyd-Jones D Adams RJ Brown TM Brown TM Carnethon M Dai S Heart disease and stroke statistics – 2010 update: a report from the American Heart Association. Circulation (2010) 121:e46–215. 10.1161/CIRCULATIONAHA.109.192667 20019324

[70] Somers VK Dyken ME Clary MP Abboud FM. Sympathetic neural mechanisms in obstructive sleep apnea. J Clin Invest (1995) 96:1897–904. 10.1172/JCI118235 7560081 PMC185826

[71] Kuniyoshi FH Garcia-Touchard A Gami AS Romero-Corral A van der Walt C Pusalavidyasagar S Day-night variation of acute myocardial infarction in obstructive sleep apnea. J Am Coll Cardiol (2008) 52:343–6. 10.1016/j.jacc.2008.04.027 18652941 PMC2598735

[72] Gami AS Olson EJ Shen WK Wright RS Ballman KV Hodge DO Obstructive sleep apnea and the risk of sudden cardiac death: a longitudinal study of 10.701 adults. J Am Coll Cardiol (2013) 62:610–6. 10.1016/j.jacc.2013.04.080 23770166 PMC3851022

